# A Secure Storage and Sharing Scheme of Stroke Electronic Medical Records Based on Consortium Blockchain

**DOI:** 10.1155/2021/6676171

**Published:** 2021-02-01

**Authors:** Qiuli Qin, Biyuan Jin, Yanqing Liu

**Affiliations:** ^1^School of Economics and Management, Beijing Jiaotong University, Beijing 100000, China; ^2^Health Service Center, Beijing Jiaotong University, Beijing 100000, China

## Abstract

The maintenance and sharing of electronic medical records are one of the essential tasks in the medical treatment combination. Traditional cloud-based electronic medical record storage system is difficult to realize data security sharing. The tamper resistance and traceability of blockchain technology provide the possibility for the sharing of highly sensitive medical data. This paper proposes a safe sharing scheme of stroke electronic medical records based on the consortium blockchain. The scheme adopts the storage method of ciphertext of medical records stored in the cloud and index of medical records stored on the blockchain. The privacy protection mechanism proposed in this paper innovatively combines proxy reencryption and searchable encryption which supports patient pseudoidentity search. The mechanism could achieve controllable sharing of medical records and precise search. According to the organizational characteristics of the stroke medical treatment combination, this paper proposes an improved Practical Byzantine Fault Tolerance mechanism to reach a consensus between consensus nodes. Then, the proposed scheme is analyzed and evaluated from three aspects of medical record integrity, user privacy, and data security. The results show that the scheme can not only ensure the privacy of patient identity information and private key data but also resist the tampering and deletion attacks of internal and external malicious nodes on the medical record data. Therefore, the proposed scheme is conducive to the improvement of the timeliness of stroke treatment and the safe sharing of electronic medical records in stroke medical treatment combination.

## 1. Introduction

Stroke is a major chronic noncommunicable disease that seriously endangers the health of Chinese people. It is the first cause of death and disability among adults in China. It has five characteristics: high morbidity, high disability, high mortality, high recurrence, and high economic burden [1]. The burden of cerebrovascular disease in China ranks first in the world. Since stroke is an emergency, whether it can be treated in a professional stroke center within the prime time is a key factor in treatment. “Healthy China 2030 Plan” put forward a request for medical institutions above the secondary level to establish a scientific regional collaborative medical treatment system for acute cerebrovascular diseases and promote the construction of stroke centers [2]. The stroke medical treatment combination is to form a hierarchical treatment system based on the stroke center in the region and realize the standardized chronic disease management of stroke disease through the community-second-level hospital-tertiary hospital linkage and cooperation model [3]. The key role of the medical treatment combination lies in the realization of referral treatment, which involves the storage and sharing of clinical and diagnosis and treatment multimode heterogeneous data such as patient medical record homepage information and electronic medical records, which contains the standard medical and clinical data gathered by physicians [4]. The rapid identification, diagnosis, and treatment of stroke determine the prognosis to a large extent. After the higher-level hospital receives the referral patient, it is essential to obtain the patient's past medical history and physical examination, blood routine examination, vascular ultrasound examination, and other risk factors screening collected in the lower-level hospital for the treatment of patients with stroke. In addition to the above check items, the electronic medical record also contains the patient's personal privacy information, such as name, date of birth, and address. The highly sensitive feature requires that the electronic medical record can not only be able to be shared authentically and credibly among medical institutions in the medical association but also meet the access control rights of the data owner and meet the needs of privacy protection [5].

The traditional stroke medical treatment combination uses cloud storage technology to build an information-sharing platform. The advantages of cloud storage technology include fast data transmission, high storage capacity, low cost, easy access to information, and dynamic communication [6, 7]. However, centralized storage is vulnerable to single-point attacks, there is a high risk of electronic medical record data leakage and tampering, and the security, integrity, and immutability of electronic medical records cannot be guaranteed. Blockchain technology is expanding its use from the parts that can be applied to transactions that exclude intermediary agencies [8]. The development of blockchain technology provides new opportunities to solve the above problems. Blockchain relies on public key encryption and hashing mechanisms to track and record the historical transactions of data on the chain. Data copies are distributed to each participating node in the network to ensure that records will not be lost or mistakenly modified, altered, or accessed by unauthorized users. And the blockchain can reach a consensus between distributed entities without relying on a single trusted party, thereby building a shared platform with trust, reliability, and transparency.

In recent years, blockchain has received extensive attention in the medical field. Medical data storage, sharing, and privacy protection have become hot research issues for scholars. To improve the storage scalability of massive, multimode, and heterogeneous medical big data, Azaria et al. [9] proposed MedRec, a scheme based on public blockchain for electronic medical records, and designed three Ethereum smart contracts to achieve fine-grained access control for patients to medical records. However, using public blockchains to store medical data is too expensive and cannot restrict the identity of network participants. Besides public blockchains, scholars have found that consortium blockchains are more suitable for medical data storage. Zhang et al. [10] proposed a secure storage and sharing scheme for medical records based on dual-blockchain and cloud server. Medical institutions form a consortium blockchain to store medical metadata. Each institution deploys a private chain to store digital abstracts of records. However, the cost of the dual-chain solution is high, and there is no guarantee that each medical institution has the economic and technical capabilities to build their own private chain. Kumar et al. [11] proposed a distributed medical data chain storage system that combines the IPFS system and blockchain technology. The scheme stores the index of medical records in the blockchain and the medical records in the IPFS distributed storage system. Research on the privacy protection of medical data based on blockchain is mainly focused on two aspects: cryptography and access control. In the field of cryptography, [12, 13] proposed medical record sharing schemes based on blockchain that use a symmetric key to encrypt medical data and then use the patient's public key to encrypt the symmetric key. In the field of access control, [14–16] proposed to use an identity-based access control model for electronic medical record sharing. The role determines the rights of the data resource. However, this method has drawbacks in fine-grained control. To solve the problem, scholars combine cryptography and access control schemes to achieve fine-grained access control. The BPDS model designed by Liu et al. [17] uses a joint scheme of CP-ABE-based access control mechanism and content extraction to achieve fine-grained access control. Niu et al. [18] proposed a secure searchable electronic medical record sharing scheme based on blockchain, which supports multiuser search and uses the ciphertext-based attribute encryption mechanism to achieve fine-grained access control. Luo et al. [19] proposed to combine distributed key generation technology with identity-based proxy reencryption technology and selected proxy nodes in the blockchain to reencrypt EHR to ensure the correctness of user private key generation. It can be found that most of the existing solutions are applied to the scenarios of patient life-cycle electronic medical record management and clinical scientific research sharing, which storage and access control methods are not suitable for single-disease electronic medical record sharing in regional referral scenarios.

Aiming at the problems of centralized storage of electronic medical records, weak interoperability, and difficulty in safe sharing in the traditional stroke medical treatment combination, this paper proposes a blockchain-based electronic stroke sharing scheme in referral scenarios to achieve targeted and accurate referrals. To meet the storage requirements, the scheme only uses the consortium blockchain for access control. The consortium blockchain stores medical metadata blocks containing medical record hash values, indexes, hospital signatures, and other information, while the original data of electronic medical records is encrypted and randomly stored in the cloud, thereby reducing the main chain pressure. To achieve the security and privacy requirements of electronic medical records sharing, this paper proposes a blockchain data privacy protection mechanism based on searchable encryption and proxy reencryption and realizes the privacy protection of patients' identity by setting anonymity. Searchable encryption technology is used to realize the encryption of key data and the ciphertext search of medical records. The searchable encryption in this study allows the search for the pseudoidentity of the patient. The proxy reencryption technology is used to realize the decryption of the requested data by the data visitor without revealing the patient's private key. Patients can designate the hospital entity to access the authorized personal data within a given time frame. In the scheme in this paper, all interactions between users and users and the blockchain are encrypted with digital signatures, and identity verification is performed to ensure system security. Aiming at the application scenario of the stroke medical treatment combination, this paper proposes to improve the preselection node rules of the Practical Byzantine Fault Tolerance (PBFT) consensus mechanism according to the classification of medical institutions in the medical treatment combination to improve the reliability and safety of the system.

## 2. Materials and Methods

### 2.1. Blockchain Technology

**Figure 1:**
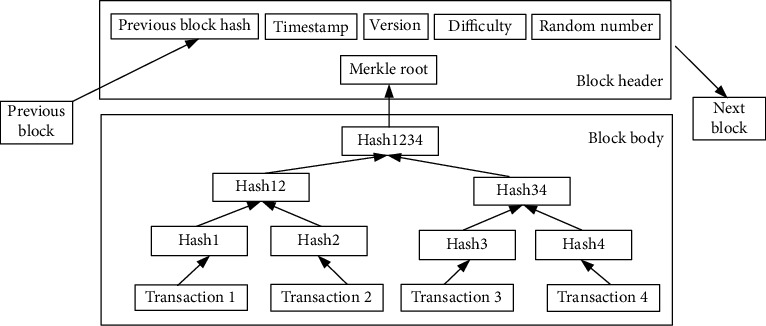
Structure of block.

Blockchain is a decentralized distributed data ledger connected by a series of ordered blocks. Nakamoto first promoted the blockchain technology that integrates cryptography and peer-to-peer communication technology in the Bitcoin white paper [20]. Blockchain is usually managed cooperatively by a peer-to-peer network, which reaches consensus by following an agreement for authenticating new blocks into the blockchain. There is no fixed central node in the blockchain network. All nodes in the network store a copy of the blockchain information. The data on a single node is tampered with or destroyed, which will not affect the data stored on the blockchain. Each block in the blockchain ledger is linked by a cryptographic hash value, that is, each block contains the hash value of the previous block content, and the irreversible hash function is used as the link mechanism to verify the integrity of the previous block [16]. This feature makes the blockchain as a decentralized distributed database immutable and traceable. The typical block structure of the Bitcoin is shown in Figure 1. A block is mainly composed of a block header and block body. The block header is made up of six components. Block header contains the hash of the previous block, the Merkle root hash value generated by the transaction ID, the timestamp when the block was generated, the version which indicates the validation rules to follow for a particular data type, and the target difficulty and random number used for consensus node to execute consensus mechanism. All the transaction orders are saved in the block body. The blocks are connected by the previous hash value to become a chain. The structure of the block ensures that the block content could not be modified or tampered with.

Blockchain networks can be divided into public chain, private chain, and consortium blockchain according to the scope of the network. The public chain is an open blockchain that allows any user to participate, and there is no identity authentication and permission setting. The transactions on the chain are completely open and transparent. All users can obtain a complete account book in the chain. Typical public chain platforms include Bitcoin and Ethereum [21]. The consortium blockchain stipulates that only approved network members can join, and it is usually managed by several institutions or organizations, and the processing speed is faster than that of the public chain. Private chains have centralized ownership and management rights and are only used within the organization. The blockchain system in the medical treatment combination scenario requires multilevel medical institutions, patients, doctors, and other entities to interact with each other, and medical record data is only accessible within the medical treatment combination, so the consortium chain is selected as the blockchain type of this paper.

### 2.2. Searchable Encryption

The searchable encryption originated from the development of cloud storage. In the cloud storage mode, cheap computing and considerable capacity attract increasingly users to outsource private data to cloud servers to save local storage and maintenance costs. However, considering the centralized characteristics of the cloud environment and the semitrusted and semihonest attributes of cloud servers, data is vulnerable to theft and loss. This problem can be solved by encrypting the data before uploading, but at the same time, there will be difficulties in ciphertext retrieval. In 2000, Song [22] first proposed the concept of searchable encryption to realize the search for ciphertext keywords without revealing user privacy. Therefore, in the medical record sharing system with cloud chain and storage proposed in this paper, searchable encryption technology is introduced, which allows searching for patients' pseudoidentities and medical record keywords to achieve precise matching.

Searchable encryption technology can be divided into symmetric searchable encryption (SSE) and asymmetric searchable encryption (ASE) according to the encryption method. SSE uses the same key for encryption and decryption, and the data owner does not need to interact with the data requester. ASE, that is, public key searchable encryption, is used to solve the problem of untrustworthy server routing. The encryption process involves two keys. The public key is used to encrypt the matching target ciphertext of the plaintext keyword information, and the private key is used to generate keyword trapdoors. In the searchable encryption mechanism, the keyword trapdoor controls the search matching behavior. Once the blockchain node receives the search trapdoor, it can perform search matching. In the medical treatment combination referral scenario, the patient has the right to use and ownership of the data, and the keyword trapdoor should be generated by the patient using private key encryption. Therefore, the scheme proposed adopts the asymmetric searchable encryption mechanism to prevent visitors from using searchable encryption public keys to generate the desired keyword trapdoors infinitely under the symmetric searchable encryption mechanism. The public-key encryption with keyword search (PEKS) algorithm first proposed by Boneh et al. [23] is described as follows:

1. *Initialization Algorithm*. Shown as Equation (1). Enter the security parameters 1^*λ*^ to obtain the private key sk and public key pk

(1) $$\begin{array}{c} \text{Setup}\left(1^{\lambda }\right)⟶\left(\text{sk},\text{pk}\right). \end{array}$$

(2) *Keyword Encryption Algorithm*. Shown as Equation (2). Enter the public key pk and keywords *w* of documents, and output the ciphertext of document keywords *c*

(2) $$\begin{array}{c} \text{Encrypt}\left(\text{pk},w\right)⟶c. \end{array}$$

(3) *Trapdoor Generation Algorithm*. Shown as Equation (3). Enter the private key sk and search keyword *w*, and output the trapdoor td corresponding to the search keyword

(3) $$\begin{array}{c} \text{Tropdoor}\left(\text{sk},w\right)⟶\text{td}. \end{array}$$

(4) *Matching Algorithm*. Shown as Equation (4). Input the generated public key pk, trapdoor td, and ciphertext *c*, and output Boolean variable *b*. When the trapdoor and ciphertext correspond to the same keyword, *b* = 1, otherwise, *b* = 0

(4) $$\begin{array}{c} \text{Test}\left(\text{td},c,\text{pk}\right)⟶b. \end{array}$$

### 2.3. Proxy Reencryption

Proxy reencryption is a cryptographic concept proposed by Blaze et al. in 1998 [24]. It is a mechanism for converting ciphertexts, which solves the problem of sharing the private key of the data owner when transferring encrypted records between nodes. A trusted third party or a semihonest agent is usually entrusted as an agent to reconstruct the encrypted message in some way. Even if another user did not encrypt the message with his associated public key, he can still use his private key to decrypt the message. Agent reencryption can ensure that even if the agent has the conversion key, he cannot obtain the plaintext information, thereby enhancing the reliability and security of the data. Proxy reencryption can be divided into one-way proxy reencryption and two-way proxy reencryption according to the direction of ciphertext conversion; according to the number of times of reencryption key conversion, it can be divided into single-hop ciphertext conversion and multihop ciphertext conversion [25]. In this paper, one-way one-hop proxy reencryption technology is adopted to ensure the privacy of the patient's private key during the medical record sharing process and reduce the number of user interactions. The master node of the consortium blockchain acts as an agent to reencrypt the ciphertext of the medical record. The workflow of proxy reencryption is as follows:

1. *Data Owner*. Encrypt the plaintext *M* with his own public key PK_*a*_ to form a ciphertext *C*_pka_, where *M* is the file that the data owner wants to give to the data requester
2. *Data Owner*. The reencryption key RK_*a*→*b*_ is formed by encrypting and calculating his own public key PK_*a*_ and the public key of the data requester PK_*b*_
3. *Agent*. Use the key RK_*a*→*b*_ generated by the data owner to convert the ciphertext *C*_pka_ into the ciphertext *C*_pkb_ that can be decrypted by the private key of the data requester and forward it to the data requester
4. *Data Requester*. Use the personal public key PK_*b*_ to decrypt the plaintext *M*

## 3. Results

To promote the information construction of the three-level diagnosis and treatment model of stroke medical treatment combination and to meet the need for doctors to quickly obtain patients' past medical history in stroke referral scenarios, this paper designs a blockchain-based stroke electronic medical record model. The purpose of the model construction includes (a) clarify the patient's ownership of medical record data and realize strict access control rights of patients to medical record data, (b) realize the privacy protection of patient identity and medical record data in the process of storage and sharing, (c) construct an efficient and secure sharing protocol to reduce user redundant operation, and (d) realize the secure storage of massive high-privacy electronic medical record data.

### 3.1. System Model

**Figure 2:**
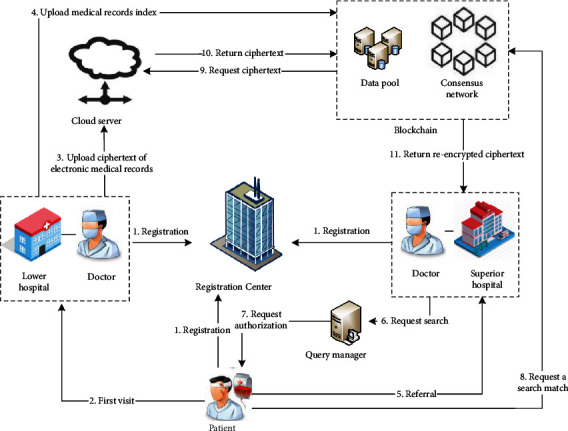
Blockchain-based sharing model for stroke electronic medical record.

The stroke electronic medical record sharing model based on the medical treatment combination referral treatment scenario proposed in this paper is displayed in Figure 2, which describes how the proposed scheme is used to store and share electronic medical records and how the entities interact to implement each phase of the scheme. The scheme contains six entities, which are registration center, query manager, patient, medical institution, consortium blockchain, and cloud server. These entities interact with each other to provide data control and protection service in the exchange of medical record information. There are four phases in the scheme proposed, user registration, data storage, request access, and request processing. In Figure 2, interaction 1 and interaction 2 are the user registration phase. Interactions 3 and 4 are the data storage phase, interactions 5, 6, 7, and 8 are the request access phase, and interactions 9, 10, and 11 are the request processing interaction.

1. *Registration Center*. The entity is used to generate and store keys. The registration center is responsible for generating the public parameters of the system, that is, the master key when the system is initialized. In addition, when a user sends a request to join the blockchain to the registration center, the entity generates a corresponding key for the user requesting registration and sends it to the user through a secure channel to authenticate the user as a legitimate member of the system. Users' identity materials will be safely stored in the registration center, and the SHA256 hash of the public key will be transmitted to the consortium blockchain for backup.

(2) *Query Manager*. The entity receives a request and authenticates the user to determine that he is a legitimate user of the system. Meanwhile, the entity formats the request in a standard manner and then forwards it to other users or the consortium blockchain network.

(3) *Patient*. Patients should register in the system when they first visit the hospital for stroke, and the registration center allocates a public-private key pair for encrypting electronic medical records and a symmetric key for generating pseudonyms for each patient. During consultation and treatment, doctors generate pseudoidentities and electronic medical records for patients. When a patient goes to a higher-level hospital for referral treatment and needs to provide the hospital with his past electronic medical records, the patient authorizes the doctor to visit, generates a search trapdoor and reencryption key for the doctor, and sends them to the master node of consortium blockchain to request a search match. In the scheme, the right to use personal medical data is completely controlled by patients. Patients can grant doctors access to relevant data, set the time limit for record access, and revoke his authorization at any time.

(4) *Medical Institution*. The entity is responsible for uploading electronic medical records and is subject to the supervision and management of the regulatory authorities. When medical institutions are registered in the blockchain, strict audit standards are required. The client of each medical institution is operated by doctors to create electronic medical records for patients. And then doctors encrypt, sign, and finally send the records to the cloud server. Medical record indexes and abstracts are sent to the consortium blockchain for storage on the chain. When a doctor at a higher-level hospital requests a medical record search, he needs to be authenticated to get patient authorization.

(5) *Cloud Server*. Due to the practical limitations of cost, storage capacity, and other factors, large-scale medical data is encrypted and stored outside the blockchain. The cloud server is used to store the ciphertext of electronic medical records uploaded by the doctor from the client, thereby reducing the storage pressure of the blockchain. When the user requests to search for data, the cloud server interacts with the blockchain. After receiving the ciphertext request from the blockchain, the ciphertext of the electronic medical record is returned to the blockchain master node for proxy reencryption.

(6) *Consortium Blockchain*. The consortium blockchain network is composed of nodes of various medical institutions. The stroke medical treatment combination includes multiple levels of medical institutions, including tertiary hospitals, second-level hospitals, and community hospitals. Each hospital acts as a node with different functions in the consortium blockchain network according to the level. They receive data requests and process them by assisting the query manager to verify the request. The consortium blockchain network uses an improved Practical Byzantine Fault Tolerant (PBFT) consensus mechanism to reach consensus among the nodes and add transaction orders to the blockchain distributed ledger. In the process of sharing medical records, after receiving the search trapdoor sent by the patient, the main node of the consortium blockchain performs search matching and sends a ciphertext request to the cloud server according to the matched index address. After receiving the ciphertext data returned by the cloud server and the reencryption key transmitted by the patient, the consortium chain master node acts as an agent to reencrypt the ciphertext data and convert it into a ciphertext that the doctor user can decrypt with the private key.

### 3.2. Scheme Phase

#### 3.2.1. User Registration

**Figure 3:**
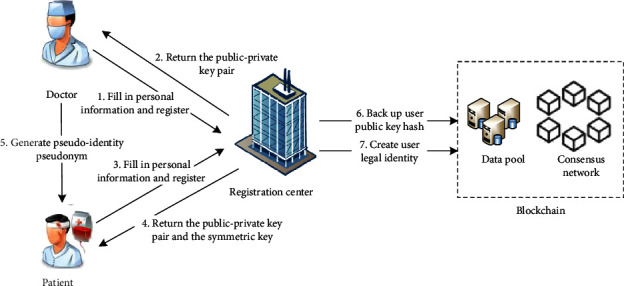
User registration.

The user registration flow chart is shown in Figure 3. A user sends a request to join the blockchain network from the client to the registration center. The registration center generates the corresponding keys for the entity and sends them to the user through a secure channel. The registration center generates a unique identification code ID for medical institutions in the consortium blockchain network, generates a public-private key pair for signatures for doctors, and generates a public-private key pair for encrypting the original data of electronic medical records and a symmetric key for generating pseudonyms for patients. When the patient visits a doctor, the doctor generates a pseudoidentity for the patient. The user's identity material will be safely stored in the registration center, and the SHA256 hash of the public key will be transmitted to the blockchain for backup, authenticating the user as a legal member of the system. Considering that there is a situation where the patient is seriously ill and cannot operate the system, this scheme allows the family members of the patient to act as agents.

#### 3.2.2. Data Storage

**Figure 4:**
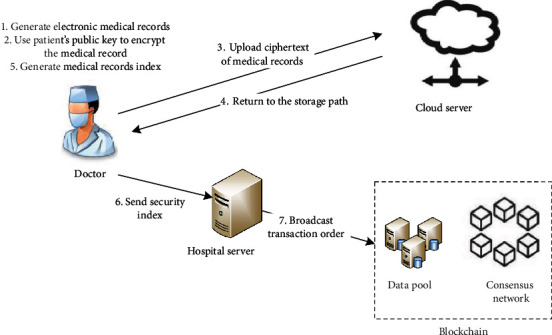
Data storage.

The data storage flow chart is shown in Figure 4. The electronic medical records storage is divided into two steps: on-chain storage and off-chain storage. First, when a patient first visits a lower-level hospital, the attending doctor A generates an electronic medical record, then encrypts it with the patient's public key, and attaches it to the signature of his private key, and transmits the record to the cloud server for storage. After receiving the storage address returned by the cloud server, doctor A extracts the medical record keywords, the pseudoidentity pseudonym of the patient, and the IP storage address of the medical record in the cloud, and then executes a searchable encryption algorithm to generate a secure index. Doctor A combines the index, his signature, personal public key, and other information to form a transaction order and sends it to the consortium blockchain for broadcasting. The node network completes the transaction on the chain through the improved PBFT consensus mechanism and realizes the data synchronization between the nodes in the chain. Doctor A who generated the medical record has read and written authority to the medical record, and there is no need to obtain a request from the patient during access.

#### 3.2.3. Request Access

**Figure 5:**
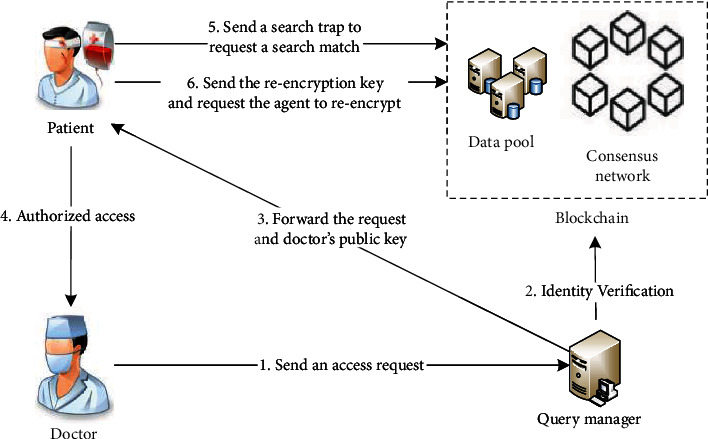
Request access.

Request access flow chart is shown in Figure 5. When a patient is referred to a higher-level hospital, the attending doctor B sends a medical record access request signed with his private key from the client. After the query manager receives the message, it combines with the blockchain to perform hybrid verification. The query manager retrieves the doctor's public key from the blockchain and verifies the signature on the request. If the signature verification is successful, the query manager forwards the access request and doctor B's public key to the patient, and the patient authorizes doctor B to access the medical records of the lower-level hospital and sets the access period. If the patient has been treated in a community hospital and a second-level hospital and referred to a third-level hospital, the doctors in the third-level hospital should be granted access to the medical records of the two lower-level hospitals. Subsequently, the patient executes an encryption algorithm to generate a search trapdoor and uses his private key and doctor B's public key to generate a reencryption key. The patient signs and packages the trapdoor, reencryption key, and doctor's request and then sends them to the master node of the consortium blockchain for search matching and proxy reencryption. The master node verifies the requester's authority and executes the next stage of request processing operations.

#### 3.2.4. Request Processing

**Figure 6:**
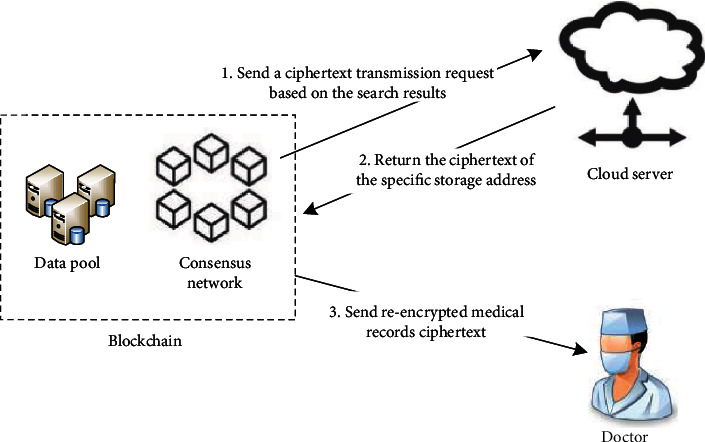
Request processing.

Request processing flow chart is shown in Figure 6. The master node of the consortium blockchain is responsible for processing the request made by system users. After receiving the search trapdoor and visit request, the master node executes the matching algorithm and sends a data request containing the specified IP storage address to the cloud server according to the search result. After receiving the returned medical record ciphertext, the master node performs a two-step verification process. First, it verifies whether the medical record returned has been authorized to be accessed by doctor B. If authorized, it verifies whether the medical record is complete. The master node hashes the ciphertext of the medical record. If the value matches the digital digest in the block transaction sheet, then it is confirmed that the medical record file has not been tampered with, and the reencryption operation can be performed. Then, the master node uses the proxy reencryption key to reencrypt the verified ciphertext of the medical record and transmit it to doctor B who requests access. When the patient has finished treatment in a tertiary hospital, he can choose to withdraw doctor B's permission to view the medical records of the lower-level hospital. The details of all transactions are formed into blocks and transmitted to the consortium blockchain.

### 3.3. System Consensus Mechanism

Practical Byzantine Fault Tolerance (PBFT) algorithm, as a state machine copy replication algorithm, can provide (*n* − 1)/3 fault tolerance (*n* is the total number of nodes in the blockchain network). The mechanism can not only start and run on fewer nodes but also does not require a lot of computing power to maintain. Considering that the number of medical institutions in the medical treatment combination is small, compared with Proof of Work (POW), Proof of Stake (POS), and Delegated Proof of Stake (DPOS) that need to rely on tokens, the PBFT mechanism is more suitable for the application scenarios of the medical treatment combination blockchain.

**Figure 7:**
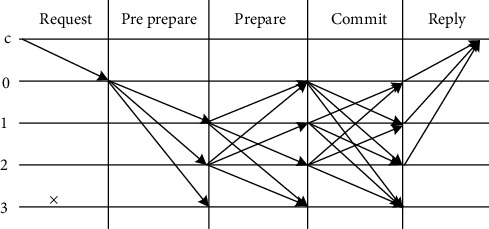
PBFT consensus mechanism.

The consortium blockchain node in this scheme is composed of medical institutions in the medical treatment combination, which is divided into two types of functional nodes according to the hospital level. High-level medical institutions such as tertiary hospitals and secondary hospitals will serve as accounting nodes due to their high server computing capabilities. Accounting nodes package the requests submitted by the medical institutions into medical data blocks and sign them with their own private keys. Small hospitals such as community hospitals are used as verification nodes to verify data blocks submitted to the consortium blockchain network and supervise behaviors related to medical data blocks. Each node reaches a consensus agreement through the improved PBFT consensus mechanism. According to the actual needs of the medical treatment combination, the improved PBFT mechanism based on the original PBFT mechanism changes the selection method of the main node from the whole network selection to fixed node rotation to improve the reliability and safety of the system. After the implementation of the improved mechanism, the secondary and tertiary hospital nodes in the medical treatment combination take turns as the master node, responsible for registering and storing data blocks in the blockchain. The improved PBFT principle is shown in Figure 7.

The improved PBFT algorithm includes five processes: request, prepreparation, preparation, confirmation, and reply. The description of each process is as follows.

1. *Request*. When the doctor uploads the medical record metadata to the blockchain, he sends a signed data upload request message from the client to the master node of the consortium blockchain and submits his personal public key as an identifier.

(2) *Prepreparation*. To verify whether the signature in the request message is correct, the master node extracts the doctor's public key, decrypts the doctor's signature in the transaction sheet to obtain the ciphertext hash value of the electronic medical record, and compares it with the digital abstract value in the transaction sheet. If the comparison results are consistent, it means that the electronic medical records are stored completely and have not been forged or tampered with. After successful verification, the master node numbers the request message and broadcasts it to all slave node members.

(3) *Preparation*. After receiving the message verification request sent by the master node, the slave node needs to pass the verification and broadcast the preparation message to other nodes except itself. If the comparison result is different, it means that the data has been tampered with, and the node will not broadcast the preparation message.

(4) *Confirmation*. The slave node sends a confirmation message to all nodes except itself and enters the reply stage after receiving 2*f* + 1 confirmation messages including itself.

(5) *Reply*. The node sends a reply message to the doctor. Only when *f* + 1 nodes receive the reply message, the request is considered executed successfully. If it is less than *f* + 1, the verification failure result will return to the user. The node checks the number of transactions in the block every one minute. If the number reaches the specified number, the node needs to form a data block and calculates the Merkle root of the block. When the specified time is reached, the node anchors the Merkle roots of all newly generated blocks to the blockchain.

### 3.4. Block Structure

**Figure 8:**
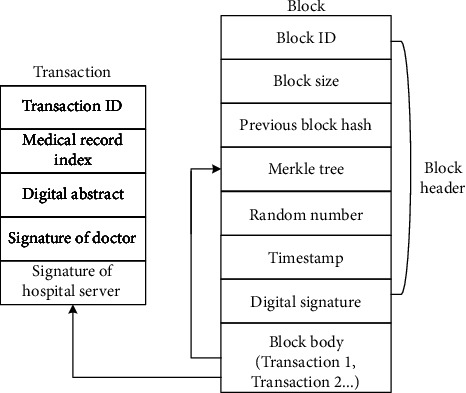
Block structure of medical consortium blockchain.

The block of the consortium blockchain proposed in this paper is composed of block header and block body. The block structure is shown in Figure 8. The block header includes block ID, block size, hash of the previous block, hash value of Merkle tree root, timestamp, and digital signature. The root hash value of the Merkle tree is the SHA256 hash value of the transaction content to ensure that the transaction sheet has not been modified. The digital signature is the signature of the block producer, confirming that the block has passed the verification; the timestamp is used to display the time when the block was generated. The content of the block body is the transaction information of the block, including transaction ID, medical record index, digital abstract, doctor's signature, and hospital server's signature. The doctor's signature refers to the digital signature information of the medical staff who generates the electronic medical record for the patient, which is used to ensure that the data can achieve accountability. The digital abstract is the hash value of the ciphertext of the medical record, which is used to ensure that the files stored in the distributed ledger cannot be forged and tampered with. The doctor's public key is the asymmetric encryption public key of the doctor who generates the electronic medical record for the patient and is used to decrypt the digital signature for block verification. The medical record index is generated by an encryption algorithm, and the information includes the IP address of the medical record in the cloud server, medical record keywords, and patient pseudoidentity ID.

### 3.5. Sharing Protocol

The sharing protocol proposed in this paper consists of four stages: system initialization, user registration, data upload, and data sharing.  Table 1 describes the symbols and their meanings in the protocol.

#### 3.5.1. System Initialization

The system initialization algorithm is shown as Equation (5). The algorithm is executed by the registration center. The registration center enters the security parameter *λ* and selects two multiplicative cyclic groups *G*_0_ and *G*_1_ with prime order *p*, *g* is the generator of *G*_0_, and then defines a bilinear mapping, *e* : *G*_0_∗*G*_0_⟶*G*_1_. Finally, the equation outputs the system public parameters PP and the system master private key MSK.

(5) $$\begin{array}{c} \left(\text{PP},\text{MSK}\right)=\text{Setup}\left(1^{\lambda }\right). \end{array}$$

#### 3.5.2. User Registration

1. *User Key Generation*. The algorithm is shown as Equation (6). It is executed by the registration center. The registration center inputs the public parameters PP, the master private key MSK, and outputs the public and private key pair *P*_ka_, *S*_ka_ assigned to the patient, the symmetric key SK_aes_ used to generate pseudonyms, and the public and private key pair *P*_*ki*_, *S*_*ki*_ assigned to the doctor for signature. After the user requests registration and completes the personal information on the system client, the registration center sends the key to the user client through a secure channel, and at the same time hashes the SHA256 of the public key and sends it to the blockchain for copy storage

(6) $$\begin{array}{c} \left(P_{k},M_{k}\right)=\text{KeyGen}\left(\text{PP},\text{MSK}\right). \end{array}$$

(2) *Searchable Encryption Key Generation*. The algorithm is shown as Equation (7). It is executed by the patient. The patient enters the security parameter *λ* and outputs the searchable encrypted public key *P*_*k*_peks_ and the searchable encrypted private key *S*_*k*_peks_. Then, the patient uploads the searchable encrypted public key *P*_*k*_peks_ to the registration center for storage, and the copy is transmitted to the blockchain for backup

(7) $$\begin{array}{c} \left(P_{k\text{\_peks}},S_{k\text{\_peks}}\right)=\text{KeyGen}\left(\lambda \right). \end{array}$$

(3) *Pseudoidentity Generation*. The algorithm is shown as Equation (8). It is executed by the doctor. The patient encrypts the symmetric key with the doctor's public key and transmits it to his doctor, and the doctor uses the private key to decrypt the symmetric key. The doctor enters the patient's symmetric key SK_aes_ and the identity code UID generated by the hospital server when the patient is registered and performs a series hash operation to output the pseudoidentity pseudonym of the patient

(8) $$\begin{array}{c} {\text{PID}}_{a}=\text{NameGen}\left({\text{SK}}_{\text{aes}},\text{UID}\right). \end{array}$$

#### 3.5.3. Data Upload

1. *Document Encryption*. The algorithm is shown as Equation (9). It is executed by the doctor. Doctor inputs the system public parameters *P*_*K*_, the plaintext document MD, the patient's public key *P*_ka_, and outputs the ciphertext medical record document EMD. And then the doctor uploads the ciphertext medical record to the cloud server, and the cloud server generates a unique ID value for each ciphertext medical record

(9) $$\begin{array}{c} \text{EMD}=\text{MDEnc}\left(P_{K},\text{MD},P_{\text{ka}}\right). \end{array}$$

(2) *Signature Generation*. The algorithm is shown as Equation (10). It is executed by the doctor. The doctor inputs the patient's ciphertext medical record document EMD and his own private key *S*_ka_ and outputs the doctor's digital signature *x*_*i*_. The encryption process is specifically to perform a hash calculation on the ciphertext to extract its digital digest and then use the doctor's private key to encrypt the digital digest to form a digital signature

(10) $$\begin{array}{c} x_{i}=\text{SignGen}\left(\text{EMD},S_{\text{ka}}\right). \end{array}$$

(3) *Index Generation*. The algorithm is shown as Equation (11). It is executed by the doctor. The doctor enters the storage path IP of the encrypted medical record EMD, the searchable encrypted public key *P*_*k*_peks_, the medical record keyword set *W*, and the patient's pseudoidentity PID_*a*_ and output the security index *I*. This process first performs a hash operation on the medical record keyword *W* and the patient's pseudoidentity PID_*a*_ to obtain *H*(*W*) and *H*(PID_*a*_) and then selects a random number *r*⟵*Z*_*p*_ to calculate the hash result and the searchable encryption public key to obtain *H*(*W*)^*r*^, *H*(PID_*a*_)^*r*^, and *P*_*k*_peks_^*r*^, and then the index *I* is calculated. The index is used for keyword search and matching in the data sharing stage

(11) $$\begin{array}{c} I=\text{IndexGen}\left(P_{k\text{\_peks}},W,{\text{PID}}_{a},\text{IP}\right). \end{array}$$

(4) *Data Upload*. Hospital server extracts the security index *I*, doctor's digital signature *x*_*i*_, and doctor's asymmetric encryption public key *P*_ki_ to construct a new transaction sheet, and adds data such as timestamp, medical record hash value, random number, and hospital's signature, and finally packs it into blocks for broadcasting

#### 3.5.4. Data Sharing

1. *Trapdoor Generation*. The algorithm is shown as Equation (12). It is executed by the patient. At the user registration stage, the patient obtains the searchable encrypted private key *S*_*k*_peks_. Patients enter the public parameter PP, the query keyword *K*_*w*_, the patient's pseudoidentity *K*_PID_, and the searchable encryption private key *S*_*k*_peks_. The algorithm first hashes the keywords or the patient's pseudoidentity, then selects a random number, and obtains the search trapdoor *T*_*w*_ after encryption calculation. The patient sends a search request to the consortium blockchain master node after generating a search transaction sheet

(12) $$\begin{array}{c} T_{w}=\text{TokenGen}\left(PP,K_{w},S_{k\text{\_peks}}\right). \end{array}$$

(2) *Search Match*. The algorithm is shown as Equation (13). It is executed by the main node of the consortium blockchain. The main node extracts the trapdoor *T*_*w*_ after receiving the search request sent by the patient and traverses the matching trapdoor *T*_*w*_ and the security index *I* of the blockchain database. If the matching is successful, the IP of the medical record containing the keyword *C*_*W*_ is obtained. The master node interacts with the cloud server and requests the cloud server to return the ciphertext of the medical record of the IP storage path

(13) $$\begin{array}{c} \text{IP}=\text{Query}\left(T_{w},I,P_{k\text{\_peks}}\right). \end{array}$$

(3) *Reencryption Key Generation*. The algorithm is shown as Equation (14). It is executed by the patient. Patient inputs his private key *S*_ka_ and the public key *P*_ki_ of the doctor requesting access, outputs the reencryption key RK, and sends it to the main node of the consortium blockchain

(14) $$\begin{array}{c} \text{RK}=\text{RKGen}\left(S_{\text{ka}},P_{\text{ki}}\right). \end{array}$$

(4) *Proxy Reencryption*. The algorithm is shown as Equation (15). It is executed by the main node of the consortium blockchain. The main node inputs the reencryption key RK and the original ciphertext medical record document EMD and outputs the reencrypted ciphertext EMD′. The reencryption process converts the EMD that needs to be decrypted with the patient's private key into EMD′, which can be directly decrypted with the doctor's private key. The master node sends EMD′ to the doctor requesting access

(15) $$\begin{array}{c} {\text{EMD}}^{\prime }=\text{ReEnc}\left(\text{RK},\text{EMD}\right). \end{array}$$

(5) *Data Decryption*. The algorithm is shown as Equation (16). It is executed by the doctor requesting access. The doctor inputs the reencrypted ciphertext EMD′ and his own private key *P*_ki_ and outputs the plaintext electronic medical record document MD

(16) $$\begin{array}{c} \text{MD}=\text{Dec}\left({\text{EMD}}^{\prime },P_{\text{ki}}\right). \end{array}$$

## 4. Discussion

### 4.1. Scheme Comparison

The paper selects the existing electronic medical record sharing scheme based on blockchain to compare with the scheme proposed in this paper and analyze the advantages and disadvantages of the schemes.  Table 2 analyzes and evaluates the selected schemes from eight aspects. Compared with the paper [14] that stores all medical electronic records on the blockchain, this paper uses cloud storage technology to reduce the pressure on blockchain storage and meet the actual deployment requirements. Compared with the DPOS consensus adopted in paper [19], the improved PBFT algorithm adopted in this paper has a low CPU occupancy rate, and it does not require a large amount of computing power to maintain. It is suitable for early exploration and later expansion of the medical blockchain. The application scenarios of this paper are similar to those in paper [26], which are sharing electronic medical records during patient referral and treatment. In this scenario, data access personnel are limited to the attending doctor, and there is no special requirement for fine-grained access control. In the process of data access, paper [27] requires the patient to transmit the private key to the doctor. The transmission process cannot guarantee the security of the private key, and it may be obtained by malicious nodes. This paper adopts the proxy reencryption method; the doctor can use the personal private key to decrypt the matched file, avoiding the possibility of private key leakage. Compared with the paper [6, 28], this paper supports the anonymity of patients and supports the search for anonymous pseudoidentities. Through accurate retrieval, it can quickly match medical records and resist node guessing attacks, thereby improving user identity security. Through comparison, it is found that this paper can be improved in the application of smart contracts. In the future work, we will consider the introduction of smart contracts to automatically execute the processing and response to requests, thereby improving transaction efficiency.

### 4.2. Scheme Analysis and Evaluation

This section analyzes whether the proposed scheme can resist internal and external attacks from three perspectives of medical record integrity, user privacy, and data security.

#### 4.2.1. Medical Record Integrity

The scheme proposed in this paper can resist electronic medical record forgery and deletion attacks. In traditional cloud-based medical record storage schemes, the cloud itself is an untrusted third party, and electronic medical records are vulnerable to forgery and erasure attacks, which cannot be detected. In the real world, to improve process efficiency, patients usually do not need to sign electronic medical records, but instead authorize doctors to directly generate and store electronic medical records. Therefore, doctors try to forge or delete electronic medical records that have been outsourced to cloud servers to cover up their medical accidents. In the blockchain-based electronic medical record sharing scheme, the electronic medical record index generated by the doctor is integrated into the transaction of the basic blockchain, and the transaction is accompanied by the hash value of the medical record ciphertext. Any change to the original data will result in a change in the hash value, thereby ensuring that the electronic medical record is unchangeable and traceable. Suppose that doctors collude with cloud servers to replace the real documents stored in the system with forged medical records. If doctors want to modify the current medical record hashes stored in the blockchain system at the same time, they must imitate the main chain like the source chain so that the blockchain transactions containing the transaction corresponding to the hash of the forged medical record can be accepted by most nodes. Due to the considerable computing power, this scenario is almost impossible to achieve. Without modifying the corresponding medical record hash, the master node will hash the data before being accessed. According to the hash rules, hash values obtained by hashing two different files are different; therefore, the forged files will not be shared and accessible. For file tampering attacks, unless the blockchain is threatened by 51% attacks, the data stored in the blockchain is immutable. The main attributes of the blockchain ensure the correctness and completeness of the electronic medical records in the cloud server.

#### 4.2.2. User Privacy

In the scheme proposed in this paper, three ways are used to ensure that user data privacy is not violated.

The first way is to adopt the encryption scheme that combines the proxy reencryption mechanism and the searchable encryption mechanism. In the scheme, the electronic medical record is encrypted by the patient's asymmetric encryption public key, and it is randomly stored anywhere in the cloud server. The blockchain only stores the index address. Therefore, to access the plain text of the electronic medical record, the requester must obtain the patient's private key and IP storage path of medical records in the cloud. In this paper, the method of proxy reencryption is adopted in the process of medical record sharing. The master node of the consortium blockchain converts the ciphertext of the medical record encrypted with the patient's public key into the ciphertext that the doctor can decrypt with his personal private key. The sharing process will not reveal the patient's private key at all. Without the patient's private key, any ciphertext information in the blockchain network cannot be encrypted. The data storage path is encrypted and stored in the consortium blockchain. Only entities with search trapdoors generated by the patient can access the medical record storage path in the cloud. Through the searchable encryption mechanism, patients have complete control over the access rights of their electronic medical record files.

The second way is to set up the access control mechanism. Access to all medical records on the medical consortium blockchain is managed by personnel, which can prevent malicious access to medical information from the source. When a doctor sends an access request, the query manager strictly verifies the identity of the visitor and prevents unauthorized behavior. Only authenticated and authorized users can access and retrieve medical record data from a specific path.

The third way is to set up pseudoidentity pseudonyms for patients. This allows all patient data to be associated with the pseudonym generated using his symmetric key, and the generated electronic medical records will not have any connection with the patient's actual identity. Therefore, malicious nodes cannot obtain the true identity of the data owner during the data sharing process. Since the pseudoidentity pseudonym generated by each attending doctor for the patient is random, the attacker cannot determine that multiple medical records are from the same patient, and the relationship between different electronic medical record data cannot be established, which ensures that the true identity of the patient cannot be traced.

#### 4.2.3. Data Security

The searchable encryption mechanism adopted by the scheme in this paper needs to send information including keyword indexes and search trapdoors to the blockchain. To ensure the security of the above information, the scheme process is analyzed to ensure that it can resist attacks from malicious nodes. First, in the index generation process, it is necessary to input the keywords of the medical records. The doctor selects a random number to calculate the keyword hash and uses the searchable encryption public key to independently execute the encryption algorithm for each keyword. Due to the uncertainty of random numbers, it is impossible for a malicious attacker to derive keywords from the keyword ciphertext. Secondly, when accessing data, the patient needs to send the search trapdoor to the blockchain node. During the construction of the search trapdoor, different keywords are used to encrypt different keywords, so the keywords can be hidden. During the sharing process, no plaintext information of the electronic medical record will be displayed.

## 5. Conclusions

The electronic medical record of stroke is the core information resource in the referral process, and its safe sharing will effectively promote doctors to accurately grasp the patient's condition. In response to the specific needs of stroke data sharing in the medical treatment combination referral scenario, this paper has provided a secure sharing protocol for electronic medical records based on the consortium blockchain. The proposed scheme has adopted a storage method combining cloud and consortium blockchain. Cloud server is used to store the ciphertext of the original medical records, and the blockchain saves traceable log information and medical record index. The proposed scheme has classified the medical institutions in medical treatment combination and improved the preselection node mechanism of the PBFT consensus algorithm to improve the reliability and security of the system. By setting the pseudoidentity pseudonym of the patient and adopting the searchable proxy reencryption scheme based on the public key encryption scheme to realize data privacy and keyword hiding. Finally, the paper has analyzed the integrity of medical records, user privacy, and data security. The results showed that the proposed scheme can resist the tampering attack of doctors and semitrusted cloud and guessing attacks of malicious nodes on patient identity and privacy. The above research shows that the scheme in this paper has the following advantages. The scheme has increasing flexibility and scalability in storage capacity and security in data privacy protection. Meanwhile, the scheme ensures that patients have ownership of medical records and provides patients with instant revocation of the right to access in access control. The scheme in this paper can also be applied to medical treatment combination for other diseases besides stroke. However, the scheme has not yet implemented access control for individual parts of the medical record, which will be part of future research. In consideration of improving the system execution efficiency, the next step of the research work is to introduce self-executing and self-verifying smart contracts and build a stroke consortium blockchain system to further refine the functional modules of client users.

## Figures and Tables

**Table 1:** Scheme symbol.

Symbol	Description
PP	System public parameters
MSK	System master private key
*P*_*k*_, *S*_*k*_	Public-private key pair of users
SK_aes_	Symmetric key of user
*P*_*k*_peks_, *S*_*k*_peks_	Searchable encryption public-private key pair
UID	Patient ID
MD	Plain text of electronic medical record
EMD	Ciphertext of electronic medical record
*x*_*i*_	Digital signature of doctor
*W*	Electronic medical record keyword set
PID_*a*_	Pseudoidentity
*I*	Electronic medical record security index
IP	Cloud storage path of ciphered medical records
*T*_*w*_	Search trapdoor
RK	Reencryption key
EMD′	Reencrypted ciphertext

**Table 2:** Comparison and analysis of schemes.

Properties	Pournaghi [6]	Zhang [14]	Luo [19]	Xu [26]	Chen [27]	Wang [28]	This paper
Application scenario	Personal life cycle management	Patient-oriented medical record sharing	Sharing of medical records among hospitals	Cross-domain visits	Clinical and scientific research	Sharing of medical records among hospitals	Sharing of referral medical records in medical treatment combination
Based on blockchain	Yes	Yes	Yes	Yes	Yes	Yes	Yes
Fine-grained access control	Yes	Yes	Yes	No	No	No	No
Patient anonymous	No	Yes	No	Yes	No	No	Yes
Keywords searchable	No	No	No	No	Yes	Yes	Yes
Consensus mechanism	PBFT	PBFT	DPOS	—	—	Improved PBFT	Improved PBFT
Main chain pressure	Small	Big	Small	Small	Small	Small	Small
Smart contract	Yes	No	No	Yes	Yes	No	No

## Data Availability

The data used to support the findings of this study are available from the corresponding author upon request.

## References

[1] Report on stroke prevention and treatment in China Writing Group (2020). Summary of China stroke prevention and treatment report 2019. *Journal of Chinese Cerebrovascular Diseases*.

[2] State Council of the PRC (2019). Outline of Healthy China 2030 Plan. *Chinese Cancer*.

[3] Zhang S. F., Han X., Wu D. H. (2018). The establishment of a new model of regional stroke management based on the intelligent medical consortium platform. *Journal of Fudan University (Medical Sciences)*.

[4] Martínez Monterrubio S. M., Frausto Solis J., Monroy Borja R. (2015). EMRlog method for computer security for electronic medical records with logic and data mining. *BioMed Research International*.

[5] Banciu D., Radoi M., Belloiu S. (2020). Information security awareness in Romanian public administration: an exploratory case study. *Studies in Informatics and Control*.

[6] Pournaghi S. M., Bayat M., Farjami Y. (2020). MedSBA: a novel and secure scheme to share medical data based on blockchain technology and attribute-based encryption. *Journal of Ambient Intelligence and Humanized Computing*.

[7] al-Absi A. A., al-Sammarraie N. A., Shaher Yafooz W. M., Kang D. K. (2018). Parallel MapReduce: maximizing cloud resource utilization and performance improvement using parallel execution strategies. *BioMed Research International*.

[8] Song Y. J. (2019). Blockchain-based power trading process. *Journal of System and Management Sciences*.

[9] Azaria A., Ekblaw A., Vieira T., Lippman A. MedRec: using blockchain for medical data access and permission management.

[10] Zhang L. H., Lan F., Jiang P. P., Jiang T. F. (2019). Medical record safe storage and sharing scheme based on dual blockchain. *Computer Engineering and Science*.

[11] Kumar R., Marchang N., Tripathi R. Distributed off-chain storage of patient diagnostic reports in healthcare system using IPFS and blockchain.

[12] Wang H., Zhou M. M. (2019). A safe storage model of medical information based on blockchain. *Computer Science*.

[13] Chen Y., Ding S., Xu Z., Zheng H. D., Yang S. L. (2019). Blockchain-based medical records secure storage and medical service framework. *Journal of Medical Systems*.

[14] Zhang Y. B., Cui M., Zheng L. J. (2019). Research on electronic medical record access control based on blockchain. *International Journal of Distributed Sensor Networks*.

[15] Hang L., Choi E., Kim D. H. (2019). A novel EMR integrity management based on a medical blockchain platform in hospital. *Electronics*.

[16] Tanwar S., Parekh K., Evans R. (2020). Blockchain-based electronic healthcare record system for healthcare 4.0 applications. *Journal of Information Security and Applications*.

[17] Liu J. W., Li X. L., Ye L., Zhang H., Du X., Guizani M. BPDS: a blockchain based privacy-preserving data sharing for electronic medical records.

[18] Niu S. F., Chen L. X., Wang J. F., Yu F. (2020). Electronic health record sharing scheme with searchable attribute-based encryption on blockchain. *IEEE Access*.

[19] Luo W. J., Wen S. L., Cheng Y. (2020). Blockchain-based electronic medical record sharing scheme. *Computer Applications*.

[20] Nakamoto S. (2018). Bitcoin: a peer-to-peer electronic cash system. http://bitcoin.org/bitcoin.pdf.

[21] Chukwu E., Garg L. (2020). A systematic review of blockchain in healthcare: frameworks, prototypes, and implementations. *IEEE Access*.

[22] Song D. X. Practical techniques for searches on encrypted data.

[23] Boneh D., Crescenzo G. D., Ostrovsky R., Persiano G. (2004). Public key encryption with keyword search. *Advances in Cryptology - EUROCRYPT 2004, International Conference on the Theory and Applications of Cryptographic Techniques*.

[24] Blaze M., Bleumer G., Strauss M. (1998). *Divertible protocols and atomic proxy cryptography*.

[25] Li L., Zeng Q. X., Wen Y. H., Wang S. C. (2020). Data sharing scheme based on blockchain and proxy re-encryption. *Information Security*.

[26] Xu J., Chen Z. M., Gong P., Wang K. K. (2019). A secure storage and access scheme for medical records based on blockchain network. *Computer Applications*.

[27] Chen L., Lee W. K., Chang C. C., Choo K. K. R., Zhang N. (2019). Blockchain based searchable encryption for electronic health record sharing. *Future generation computer systems*.

[28] Wang H., Liu Y. X., Cao S. Y., Zhou M. M. (2020). Medical data storage mechanism incorporating blockchain technology. *Computer Science*.

